# Hypusinated eIF5A is expressed in the pancreas and spleen of individuals with type 1 and type 2 diabetes

**DOI:** 10.1371/journal.pone.0230627

**Published:** 2020-03-24

**Authors:** Teresa L. Mastracci, Stephanie C. Colvin, Leah R. Padgett, Raghavendra G. Mirmira

**Affiliations:** 1 Indiana Biosciences Research Institute, Indianapolis, Indiana, United States of America; 2 Department of Biochemistry and Molecular Biology, Indiana University School of Medicine, Indianapolis, Indiana, United States of America; 3 Center for Diabetes and Metabolic Diseases, Indiana University School of Medicine, Indianapolis, Indiana, United States of America; 4 Department of Pediatrics, Indiana University School of Medicine, Indianapolis, Indiana, United States of America; 5 Department of Physiology, Indiana University School of Medicine, Indianapolis, Indiana, United States of America; 6 Herman B Wells Center for Pediatric Research, Indiana University School of Medicine, Indianapolis, Indiana, United States of America; Unicamillus, Saint Camillus International University of Health Sciences, ITALY

## Abstract

The gene encoding eukaryotic initiation factor 5A (*EIF5A*) is found in diabetes-susceptibility loci in mouse and human. eIF5A is the only protein known to contain hypusine (hydroxyputrescine lysine), a polyamine-derived amino acid formed post-translationally in a reaction catalyzed by deoxyhypusine synthase (DHPS). Previous studies showed pharmacologic blockade of DHPS in type 1 diabetic NOD mice and type 2 diabetic db/db mice improved glucose tolerance and preserved beta cell mass, which suggests that hypusinated eIF5A (eIF5A^Hyp^) may play a role in diabetes pathogenesis by direct action on the beta cells and/or altering the adaptive or innate immune responses. To translate these findings to human, we examined tissue from individuals with and without type 1 and type 2 diabetes to determine the expression of eIF5A^Hyp^. We detected eIF5A^Hyp^ in beta cells, exocrine cells and immune cells; however, there was also unexpected enrichment of eIF5A^Hyp^ in pancreatic polypeptide-expressing PP cells. Interestingly, the presence of eIF5A^Hyp^ co-expressing PP cells was not enhanced with disease. These data identify new aspects of eIF5A biology and highlight the need to examine human tissue to understand disease.

## Introduction

The mechanisms underlying the pathogeneses of type 1 diabetes (T1D) and type 2 diabetes (T2D) involve the activation of systemic and local inflammatory pathways, leading to eventual dysfunction, de-differentiation and/or death of the beta cells in the pancreatic islet. Elucidating the molecular mechanisms driving the inflammatory response is applicable to the development of therapies for both diseases. In addition, an urgent priority in T1D research is the discovery of biomarkers that can assist in the identification of individuals with pre-clinical disease so early preventative therapeutic interventions can be implemented.

Recently, our laboratories have been investigating the involvement of the hypusinated form of eukaryotic initiation factor 5A (eIF5A) in the development and progression of diabetes in mice. To date, eIF5A is the only known protein to contain hypusine (hydroxyputrescine lysine) [1], which is a polyamine-derived amino acid. This post-translational modification, formed by the process of “hypusination” [2], is catalyzed through a multi-step reaction initiated by the *rate-limiting* enzyme deoxyhypusine synthase (DHPS) and uses the polyamine spermidine as a cofactor to modify the Lys50 of eIF5A [2]. Previous studies using human cell lines and yeast determined that eIF5A, the hypusinated form of eIF5A (eIF5A^Hyp^) and DHPS are vital for cell viability and proliferation [3,4]. Evolutionarily, eIF5A is highly conserved including the amino acid sequence surrounding the hypusine residue, which suggests an important role for this modification [5]. Whereas studies across species have established that eIF5A^Hyp^ efficiently binds the ribosome complex and facilitates mRNA translation [3,6,7], the exact function of eIF5A and eIF5A^Hyp^ remains unknown.

Interestingly, the gene encoding eIF5A is found in the *Idd4* diabetes-susceptibility locus in non-obese diabetes (NOD) mice [8,9]. In prior studies, we showed that eIF5A^Hyp^ is expressed in the pancreatic islets of mouse [10,11], is responsible for the translation of a subset of cytokine-induced transcripts in beta cells in mouse models of diabetes [12,13], and that eIF5A^Hyp^ also appears to be required for the activation and proliferation of effector T helper cells [14]. Moreover, reducing the hypusination of eIF5A in NOD mice, a model of T1D, by pharmacological inhibition of DHPS resulted in reduced insulitis, improved glucose tolerance and preserved beta cell mass [14]. Similarly, pharmacological blockade of DHPS in db/db mice [15], a model of T2D improved glucose tolerance and enhanced beta cell mass [16]. Together these data suggest that eIF5A^Hyp^ may play a role in the pathogenesis of diabetes such that altering the expression of eIF5A^Hyp^ may improve diabetes outcomes long-term.

To translate these findings to human, a greater understanding of eIF5A^Hyp^ in the human pancreas and spleen is required. In particular, determining the expression pattern of eIF5A^Hyp^ in human tissues and whether eIF5A^Hyp^-expressing cells stratify with characteristics of disease would be informative. In this study, we used human donor tissue samples from the Network of Pancreatic Organ Donors with Diabetes (nPOD) (www.jdrfnpod.org) to examine the expression pattern of eIF5A^Hyp^ in the human pancreas and spleen from individuals with T1D, T2D and non-diabetic controls.

## Materials and methods

### Pancreas and isolated islet cells from mouse

All mice were purchased from the Jackson Laboratory and maintained under a protocol approved by the Indiana University School of Medicine Institutional Animal Care and Use Committee (OLAW Assurance Number: D16-00584 (A4091-01); USDA Certificate Number: 32-R-0025; Customer ID: 798; AAALAC Unit Number: 00083; Protocol Approval #19043). The approved method of euthanasia for mice was carbon dioxide inhalation overdose delivered using a gas cylinder, flow meter/regulator, and induction chamber; 100% CO2 delivered at a rate such that 20–30% of the volume of the chamber is displaced per minute. This was followed by the secondary method of cervical dislocation. Human donor tissues were collected and provided to the investigator by the Network of Pancreatic Organ Donors with Diabetes (https://www.jdrfnpod.org). The study was approved by the University of Florida Institutional Review Board (IRB-1); approval #IRB201600029. Written consent was obtained.

Total pancreas and isolated islets from wildtype C57BL/6 mice as well as acinar tissue and isolated islets from human donors (Table 1 details human donors) were subjected to immunoblot analysis as previously described [17]. Rabbit anti-eIF5A^Hyp^ ([13,18]; 1:1000), mouse anti-eIF5A (BD Biosciences; 1:2000) and guinea pig anti-insulin (DAKO; 1:5000) antibodies were used to confirm protein expression in the pancreas.

**Table 1:** Human donor information for islet and acinar tissue preparations.

	Islet Preparation	Acinar Preparation
Unique identifier	SAMN11578698	UNOS AGDB487
Donor Age (years)	57.0	24
Donor Sex (M/F)	M	M
Donor BMI (kg/m^2^)	25.8	31.7
Donor HbA1c	5.7	not tested
Origin/source of islets	IIDP (Integrated Islet Distribution Program)	CORE (Center for Organ Recovery and Education)
Islet isolation center	The Scharp-Lacy Research Institute	Pittsburgh (AHN)
Donor history of diabetes? Yes/No	No	No
**If Yes, complete the next two lines if this information is available**		
Diabetes duration (years)	Not Applicable	Not Applicable
Glucose-lowering therapy at time of death	Not Applicable	Not Applicable

Mice containing the *RIP-cre* allele (B6.CG-Tg(Ins2-cre)25Mgn/J) [19] were mated with those containing the *R26R*^*Tomato*^ allele (B6.Cg-Gt(ROSA)26Sor^tm14(CAG-tdTomato)Hze/J^) [20] to produce double transgenic animals wherein all the insulin-producing beta cells in the pancreas expressed a fluorescent (Tomato) reporter. Pancreatic islets were isolated from *RIP-cre;R26R*^*Tomato*^ mice and *R26R*^*Tomato*^ mice as previously described [21]. The isolated islets from all mice were pooled together and processed for fluorescence activated cell sorting (FACS), which facilitated the separation of islet cells into two populations: Tomato-positive beta cells and Tomato-negative non-beta cells (which included islet cells expressing glucagon, somatostatin, ghrelin, and pancreatic polypeptide). Pooled islets were washed with sterile PBS (Fisher Scientific) and incubated in Accutase cell detachment solution (Sigma) for 10 minutes at 37C with constant mixing (1000 rpm). Islet cells were removed from the Accutase solution by centrifugation (500 x g for 1 min) and resuspended in cold buffer containing 2% BSA,1uM EDTA, and equal parts PBS and HBSS (Fisher Scientific). The cells were filtered, collected and incubated with APC viability dye (Zombie NIR-IR dye; BioLegend) per the manufacturer’s recommended protocol. Single-cell suspensions from *RIP-cre;R26R*^*Tomato*^ mice and *R26R*^*Tomato*^ were then sorted using an iCyt Reflection with 100 μm nozzle at 23 psi. Dead cells (NIR-IR+) were excluded; Tomato(+) cells and Tomato(-) cells were collected into tubes containing sort buffer. Data were analyzed using FlowJo software (Tree Star). Lysates from the two populations of cells were subjected to immunoblot analysis. Rabbit anti-eIF5A^Hyp^ and mouse anti-eIF5A antibodies were used as above, to evaluate the abundance of eIF5A^Hyp^ in the beta cell and non-beta cell populations. Rabbit anti-Pdx1 (Chemicon; 1:1000) antibody was used to evaluate enrichment of the beta cell (tomato+) population.

### Mouse pancreas tissue and immunofluorescence analysis

Pancreas tissue was harvested from wildtype C57BL/6 mice and fixed in 4% paraformaldehyde (Fisher Scientific), cryo-preserved using 30% sucrose, embedded in OCT (Fisher Scientific) and sectioned onto glass microscope slides. Methods previously described for pancreas preservation and immunofluorescence were followed [22]. Pancreas tissue sections (8 μm) were stained using the following primary antibodies: guinea pig anti-insulin (DAKO; 1:500), goat anti-pancreatic polypeptide (abcam; 1:200), rabbit anti-eIF5A^Hyp^ ([13,18]; 1:1000). Secondary antibodies including Alexa-488, Cy3, or Alexa-647 (Jackson Immunoresearch) were used, followed by DAPI (Sigma; 1:1000) to visualize nuclei. Images were acquired with a Zeiss 710 confocal microscope.

### Human pancreas and spleen tissue

Paraffin-embedded tissue sections were obtained from the nPOD consortium (www.jdrfnpod.org). A total of 10 nondiabetic donors, 4 donors with T2D, and 12 donors with T1D (6 autoantibody positive, 6 autoantibody negative) were included in this study (Tables 2 and 3). Information regarding donors’ demography, histology, and disease status were provided by nPOD. The autoantibody status was also determined by nPOD as previously described [23].

**Table 2:** Human donor pancreas and spleen tissue from T2D and matched controls.

nPOD case #	Sample name	Age (years)	Gender (male/female)	Ethnicity	BMI	T2D (yes/no)	C-peptide (ng/mL)	HbA1c
nPOD-6097	F1-control	43.1	female	Caucasian	36.4	no	16.76	7.1
nPOD-6102	F2-control	45.1	female	Caucasian	35.1	no	0.55	6.1
nPOD-6132	F1-T2D	55.8	female	Hispanic	44.6	yes	0.8	9.1
nPOD-6109	F2-T2D	48.8	female	Hispanic	32.5	yes	<0.05	8
nPOD-6060	M1-control	24	male	Caucasian	32.7	no	13.63	N/A
nPOD-6091	M2-control	27.1	male	Caucasian	35.6	no	7.71	6.3
nPOD-6114	M1-T2D	42.8	male	Caucasian	31	yes	0.58	7.8
nPOD-6188	M2-T2D	36.1	male	Hispanic	30.6	yes	3.45	7.2

**Table 3:** Human donor pancreas and spleen tissue from T1D and matched controls.

nPOD case #	Sample name	Age (years)	Gender (male/female)	Ethnicity	BMI	T1D (yes/no)	c-peptide	Auto Antibodies Detected	Duration of disease (years)
nPOD-6179	1-control	21.8	female	Caucasian	20.7	no	2.74	N/A	N/A
nPOD-6224	1-AAb-negative	21	female	Caucasian	22.8	yes	<0.05	negative	1.5
nPOD-6070	1-AAb-positive	22.6	female	Caucasian	21.6	yes	<0.05	mIAA+;1A-2A+	7
nPOD-6034	2-control	32	female	Caucasian	25.2	no	3.15	N/A	N/A
nPOD-6121	2-AAb-negative	33.9	female	Caucasian	18	yes	0.24	negative	4
nPOD-6143	2-AAb-positive	32.6	female	Caucasian	26.1	yes	<0.05	1A-2A+;mIAA+	7
nPOD-6229	3-control	31	female	Caucasian	26.9	no	6.23	N/A	N/A
nPOD-6208	3-AAb-negative	32	female	Caucasian	23.4	yes	<0.05	negative	16
nPOD-6077	3-AAb-positive	32.9	female	Caucasian	22	yes	<0.05	mIAA+	18
nPOD-6015	4-control	39	female	Caucasian	32.2	no	1.99	N/A	N/A
nPOD-6038	4-AAb-negative	37.2	female	Caucasian	30.9	yes	0.2	negative	20
nPOD-6054	4-AAb-positive	35.1	female	Caucasian	30.4	yes	<0.05	mIAA+	30
nPOD-6055	5-control	27	male	Caucasian	22.7	no	0.59	N/A	N/A
nPOD-6041	5-AAb-negative	26.3	male	Caucasian	28.4	yes	<0.05	negative	10
nPOD-6180	5-AAb-positive	27.1	male	Caucasian	25.9	yes	<0.05	GADA+;1A-2A+; ZnT8A+;mIAA+	11
nPOD-6104	6-control	41	male	Caucasian	20.5	no	20.55	N/A	N/A
nPOD-6173	6-AAb-negative	44.1	male	Caucasian	23.9	yes	<0.05	negative	15
nPOD-6141	6-AAb-positive	36.7	male	Caucasian	26	yes	<0.05	GADA+;1A-2A+; ZnT8A+;mIAA+	28

### Immunofluorescence analysis of human tissues

Immunofluorescent staining was performed as previously published [22] with modifications to account for the use of paraffin embedded tissue. Briefly, tissue sections were deparaffinized through graded ethanols (100%, 95%, 85%, 75%, 50%; Fisher Scientific) and then blocked using normal donkey serum (Sigma). Primary antibodies used included guinea pig anti-insulin (DAKO; 1:500), mouse anti-glucagon (Abcam; 1:500), rat anti-somatostatin (abcam; 1:200), goat anti-pancreatic polypeptide (abcam; 1:200), goat anti-ghrelin (Santa Cruz; 1:500), mouse anti-Pax5 (DAKO; 1:200), mouse anti-CD8 (Thermo Fisher; 1:500), mouse anti-CD4 (Leica; 1:500), rabbit anti-eIF5A^Hyp^ ([13,18]; 1:1000). Secondary antibodies including Alexa-488, Cy3, or Alexa-647 (Jackson Immunoresearch) were used to visualize primary antibodies. DAPI (Sigma; 1:1000) was used to visualize nuclei. Images were acquired with a Zeiss 710 confocal microscope.

### Drug treatments of cultured cells and mouse islets

HEK293T cells (ATCC #CRL-3216) were maintained in Dulbecco’s modified Eagle’s medium (DMEM) supplemented with 10% FBS (Hyclone; Fisher Scientific), 1% Penicillin/Streptomycin and 2 mM L-Glutamine. HEK293T cells were grown to 60–70% confluency on coverslips in a 24-well plate and treated with 10 or 100 μM N1-Guanyl-1,7-diaminoheptane (GC7) [24] (or 10 mM acetic acid; vehicle) with 0.5 mM aminoguanidine for 16 hours. Cells were fixed with 4% paraformaldehyde in PBS and blocked for 30 min in 3% bovine serum albumin (BSA) in PBS followed by permeabilization with 0.2% Triton X-100 in BSA-PBS for 10 min. All antibodies were diluted in 3% BSA-PBS and were applied in sequential order. Cells were incubated with rabbit anti-eIF5A^Hyp^ ([13,18], 1:1000) overnight at 4°C followed by a one hour incubation with anti-rabbit 488 (Jackson Immunoresearch; 1:500) at room temperature. Coverslips were washed with PBS, and DAPI (Sigma; 1:1000) used to visualize nuclei. Coverslips were mounted and images acquired with a Zeiss 710 confocal microscope.

Mouse islets were isolated as previously described [21] and subsequently cultured in RPMI 1640 media supplemented with 10% FBS (Hyclone; Fisher Scientific), and 1% Penicillin/Streptomycin. Islets were treated with 100 μM GC7 (or 10 mM acetic acid; vehicle) with 0.5 mM aminoguanidine for 72 hours; the duration of treatment to reduce hypusination was previously determined in [10]. Islet were fixed with 4% paraformaldehyde in PBS and blocked for 1 hour in 5% normal donkey serum (NDS) in PBS followed by permeabilization with 0.1% Triton X-100 in NDS-PBS for 10 min. Islets were stained as described above using chambered slides; maximum intensity projection images were processed from Z-stack image collections acquired with a Zeiss 710 confocal microscope.

## Results

### Beta cell and non-beta cell distribution of eIF5A^Hyp^ in mouse

**Fig 1:**
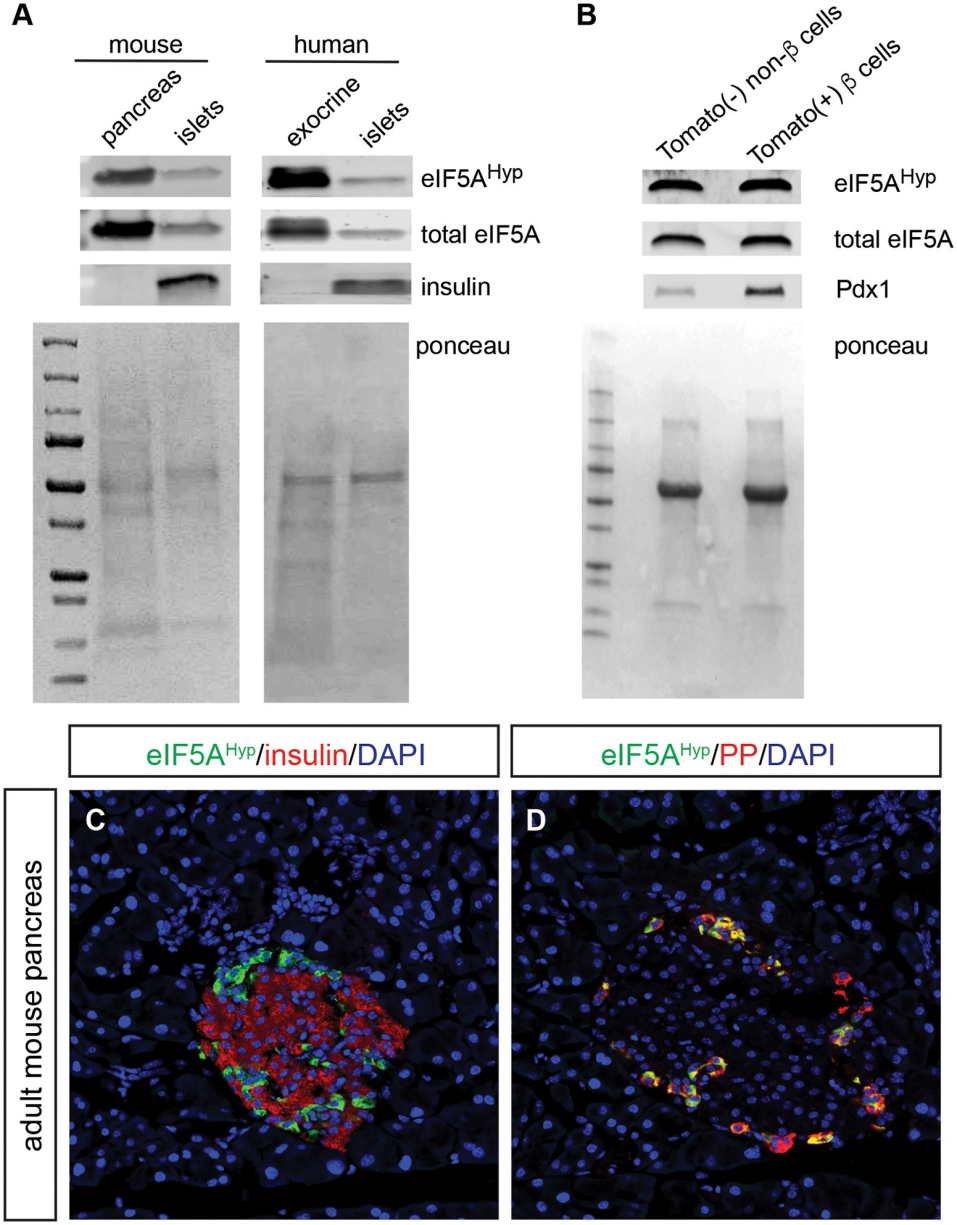
Expression of hypusinated eIF5A (eIF5A^Hyp^) in mouse and human pancreatic islets. (A) Western blot from mouse and human pancreas tissue and isolated pancreatic islets. (B) Western blot from FACS sorted mouse islet cell populations. (C, D) Representative immunofluorescence images of mouse tissue demonstrating robust expression of eIF5A^Hyp^ in PP-expressing cells.

We previously developed and characterized a novel antibody that recognizes the unique amino acid hypusine, formed exclusively through posttranslational modification of the Lys50 residue of eIF5A (eIF5A^Hyp^) [12,18]. In this study, we utilized this antibody to investigate the expression of eIF5A^Hyp^ in mouse and human pancreas tissue and isolated islets as well as human spleen tissue, to characterize the expression pattern of eIF5A^Hyp^ and determine if eIF5A^Hyp^-expressing cells stratify with characteristics of disease. To that end, we first confirmed the presence of eIF5A^Hyp^ in islets isolated from mouse and human pancreas as well as in mouse pancreas and human acinar (exocrine) tissue (Fig 1A, S1 Fig).

We next utilized the *RIP-cre;R26R*^*Tomato*^ mouse model wherein the insulin-expressing cells were labeled with a lineage trace, thereby generating beta cells indelibly marked with fluorescent reporter (Tomato) expression. Islet cells from *RIP-cre;R26R*^*Tomato*^ and control animals were sorted by FACS, using the presence and absence of Tomato expression to separate cells into two populations: beta cells (Tomato-positive) and non-beta cells (Tomato-negative). The cell types represented in the “non-beta cell” sample included (ordered from largest population to smallest): glucagon-expressing alpha cells, somatostatin-expressing delta cells, pancreatic polypeptide-expressing PP cells, ghrelin-expressing epsilon cells, exocrine cells (a possible contaminant from the process of islet isolation) and support cells including endothelial cells. A similar quantity of Tomato-positive beta cells (1.92x10^5^ cells) and Tomato-negative non-beta cells (2.13x10^5^ cells) were collected (S2 Fig). Subsequent western blot analysis identified that eIF5A^Hyp^ was present in nearly identical abundance in both the beta cell (Tomato-positive) and non-beta cell (Tomato-negative) populations (Fig 1B, S3 Fig). The expression of Pdx1 confirms the enrichment of beta cells in the Tomato positive cells; the lower level of Pdx1 expression in the non-beta cell fraction can be attributed to the presence of somatostatin-expressing delta cells. These data demonstrate that eIF5A^Hyp^ is expressed in both the beta cell and non-beta cell fractions; however, whether there is a differential expression of eIF5A^Hyp^ in a specific non-beta cell type(s) cannot be clarified from these data. Therefore, to characterize the spatial distribution of eIF5A^Hyp^ expression pattern in the islet, we performed co-immunofluorescence staining for eIF5A^Hyp^ and islet hormones in mouse pancreas tissue. Whereas relatively weak immunostaining of eIF5A^Hyp^ was found throughout the pancreas and islets, robust immunostaining of eIF5A^Hyp^ was found in the islet cell population that expressed pancreatic polypeptide (Fig 1C and 1D).

To confirm that the high expressing cell population was not an artifact and that our previously published antibody [12,18] could detect expression of eIF5A^Hyp^ by immunofluorescence, we treated HEK293T cells (human) or isolated pancreatic islets (mouse) with the DHPS inhibitor GC7 (N1-Guanyl-1,7-diaminoheptane) [24]. Cells and islets were then analyzed for eIF5A^Hyp^ by immunofluorescence. Whereas the control HEK293T uniformly expressed eIF5A^Hyp^, the mouse islets contained cells with both weak and robust expression of eIF5A^Hyp^. Furthermore, following treatment with the inhibitor, we observed a reduction in expression of eIF5A^Hyp^ in both the HEK293T cells and mouse islets compared with vehicle treated controls (S4 Fig), which verified the measurement of eIF5A^Hyp^ expression using our antibody.

### eIF5A^Hyp^-expressing cells in the pancreas of human type 2 diabetes

To characterize the expression pattern of eIF5A^Hyp^ in the human pancreas, we utilized tissue samples from the Network of Pancreatic Organ Donors with Diabetes (nPOD). A cohort of tissues from donors with and without T2D were provided (Table 2). Both pancreas and spleen tissues were acquired from each donor; age, gender, ethnicity and BMI were matched where possible. Given the relatively small size of the cohort, quantitative evaluations were not possible. Therefore, we evaluated the presence or absence of eIF5A^Hyp^, its cell-type expression pattern, and its expression correlation with disease.

**Fig 2:**
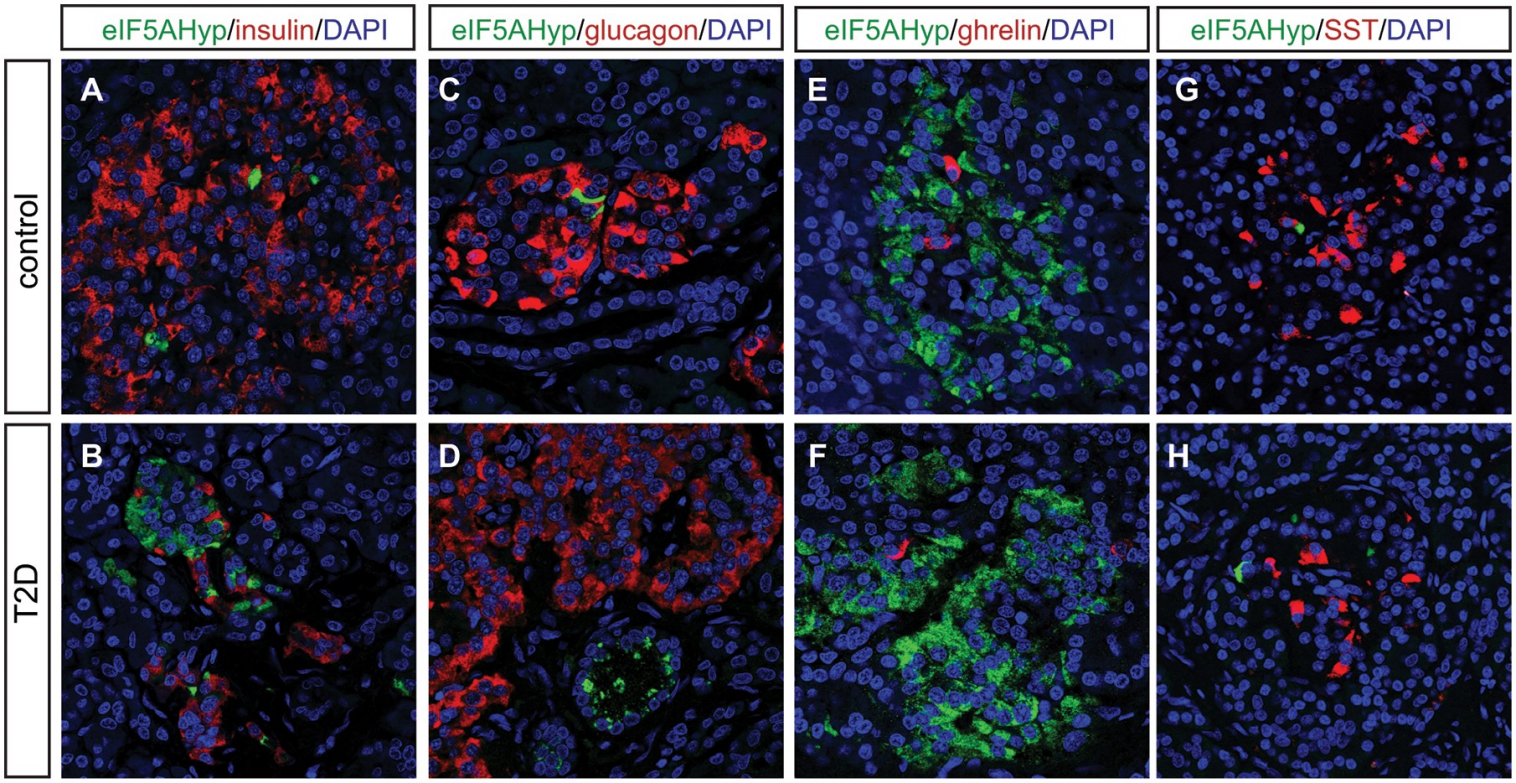
The expression pattern of eIF5A^Hyp^ in T2D and control pancreatic tissue. In controls (matched for age, gender and BMI) and T2D pancreas, we evaluated the co-expression of eIF5A^Hyp^ with all islet hormones and found no overlap with insulin (A,B), glucagon (C,D), ghrelin (E,F) or somatostatin (G,H).

**Fig 3:**
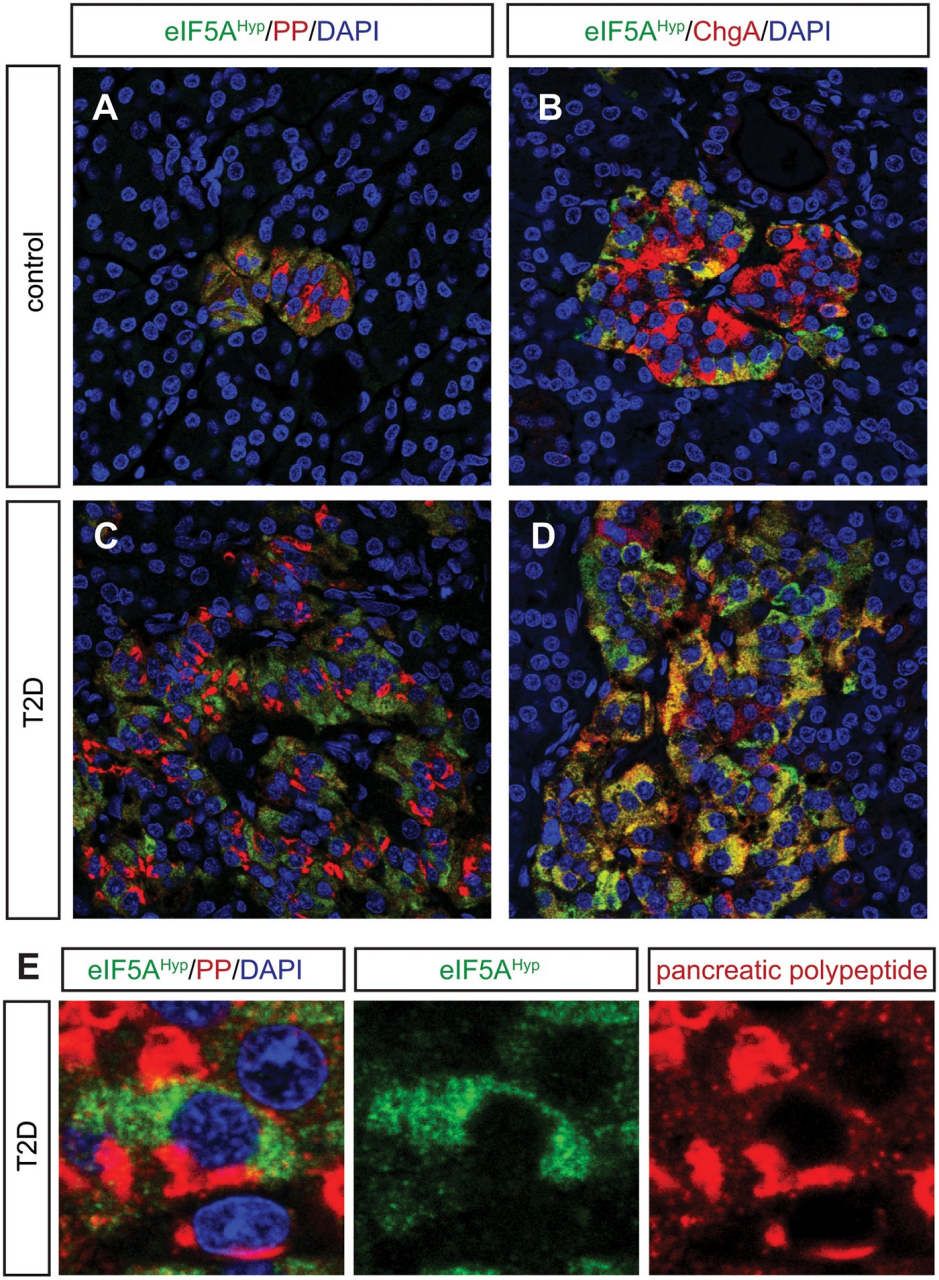
eIF5A^Hyp^ is robustly expressed in the pancreatic polypeptide-expressing PP cells in the islet. (A-D) In both controls and T2D pancreas, co-expression of eIF5A^Hyp^ with pancreatic polypeptide (PP) and chromograninA (ChgA) was observed. (E) An expression pattern of eIF5A^Hyp^ in the PP cells suggestive of localization to the ER was observed in cells in both controls and T2D pancreas.

Pancreas tissue sections were co-immunostained with the eIF5A^Hyp^-specific antibody and antibodies that recognized the hormones expressed by each of the endocrine cell populations in the islet (insulin, glucagon, somatostatin, ghrelin and pancreatic polypeptide). Robust co-localization was not observed between eIF5A^Hyp^ and insulin (Fig 2A and 2B), glucagon (Fig 2C and 2D), ghrelin (Fig 2E and 2F), or somatostatin (Fig 2G and 2H). However, as observed in the mouse pancreas, cells expressing pancreatic polypeptide were identified to co-express high levels of eIF5A^Hyp^ in control pancreas tissue (Fig 3A). These cells also expressed chromograninA, which confirms their identity as neuroendocrine cells (Fig 3B). The co-localization of eIF5A^Hyp^ with pancreatic polypeptide in the PP-expressing cells was observed in pancreas tissues from donors with T2D (Fig 3C and 3D) and non-diabetic controls, suggesting no differential expression related to disease status. Notably, whereas PP and eIF5A^Hyp^ were expressed in the same cells, the expression pattern is suggestive of localization in different compartments (Fig 3E).

**Fig 4:**
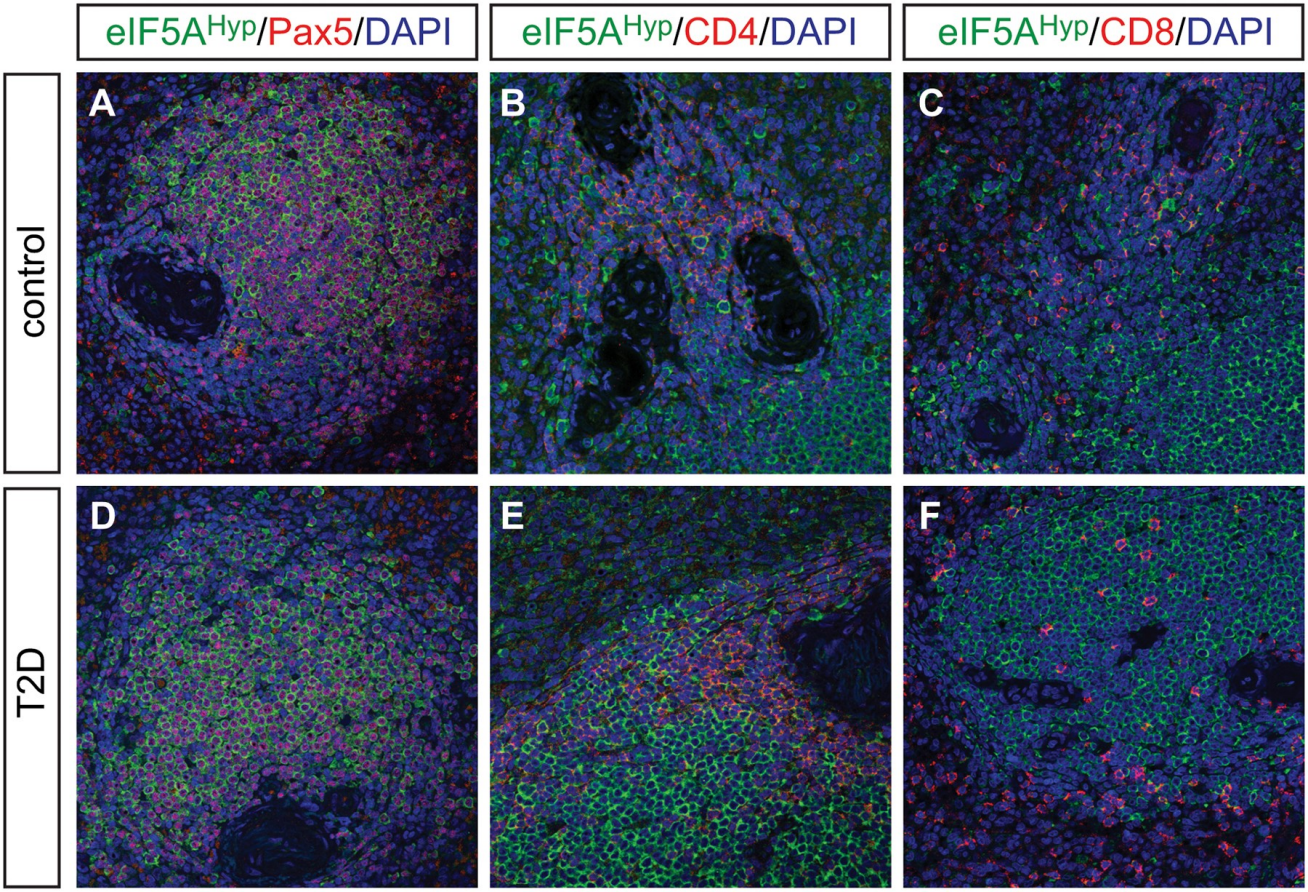
eIF5A^Hyp^ expression pattern in the spleen of control and T2D. eIF5A^Hyp^ is expressed in immune cells in the spleen. We evaluated expression of eIF5A^Hyp^ in Pax5+ B cells, CD4+ T cells and CD8+ T cells in the spleens of donors with T2D and controls matched for age, gender and BMI. Most eIF5A^Hyp^+ cells co-expressed Pax5+ (A,D); however, a select group of eIF5A^Hyp^+ cells expressed either CD4+ (B,E) or CD8+ (C,F).

Spleen tissue sections from the same donors were co-immunostained with eIF5A^Hyp^ and markers of various cell types. In particular, Pax5-expressing B cells, CD4-expressing T cells, and CD8-expressing T cells were evaluated for co-expression of eIF5A^Hyp^. Whereas the expression patterns observed suggest that most Pax5+ B cells expressed eIF5A^Hyp^, only a select group of eIF5A^Hyp^-expressing cells appear to co-expressed either CD4 or CD8 (Fig 4A–4C; S5–S7 Figs). No obvious differences in staining intensity or distribution were observed between samples from T2D and controls (Fig 4D–4E).

### eIF5A^Hyp^-expressing cells in the pancreas of human type 1 diabetes

**Fig 5:**
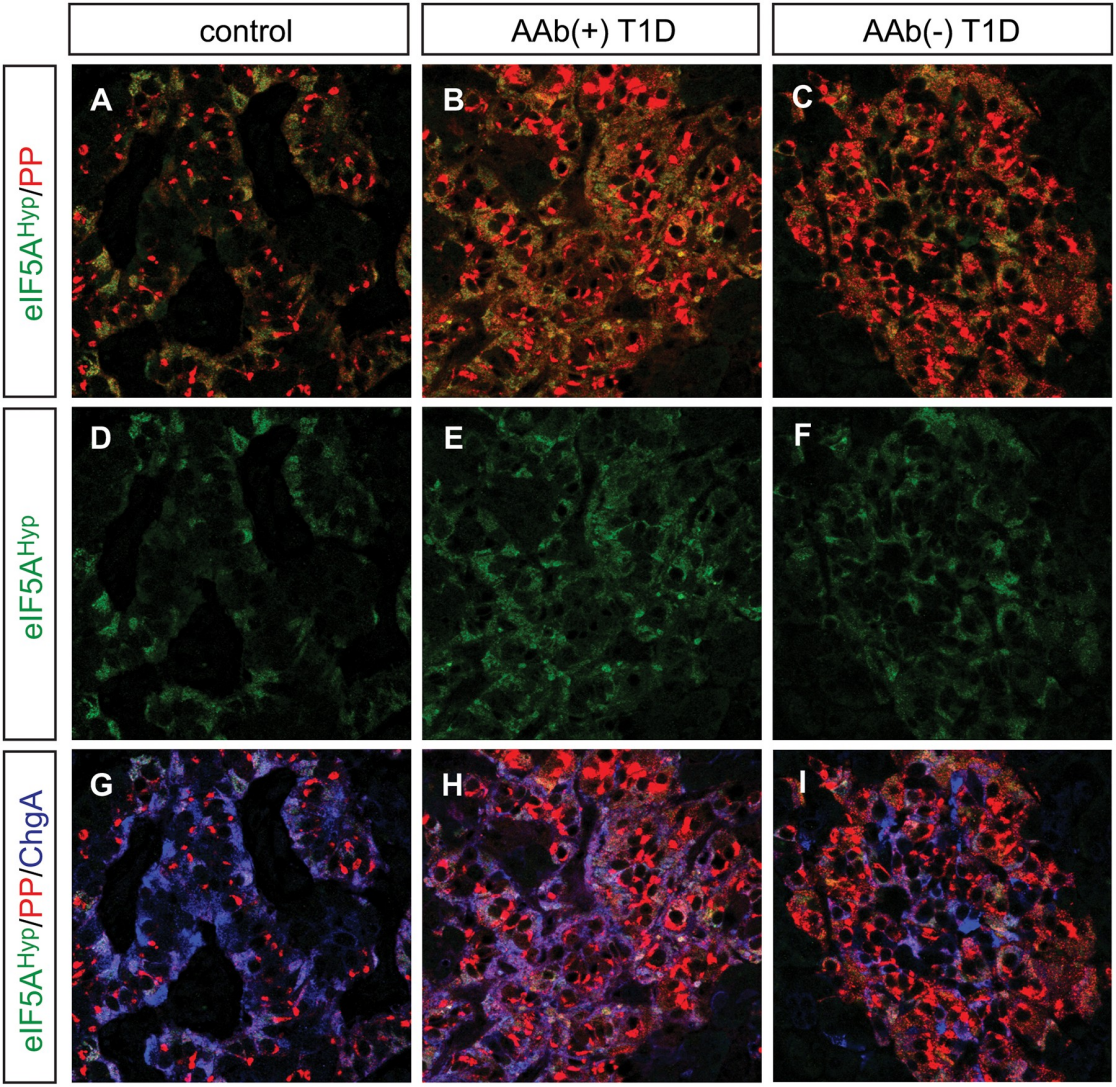
Expression of eIF5A^Hyp^ in the T1D pancreas. (A-F) Identical to the pattern identified in T2D and control tissues, high expression of eIF5A^Hyp^ is observed in PP cells in the T1D, both auto-antibody positive (aAb+) and auto-antibody negative(aAb-), pancreas and controls (matched for age, gender, ethnicity and BMI). (G-I) In all cases, these cells express the endocrine cell marker ChromograninA (ChgA).

**Fig 6:**
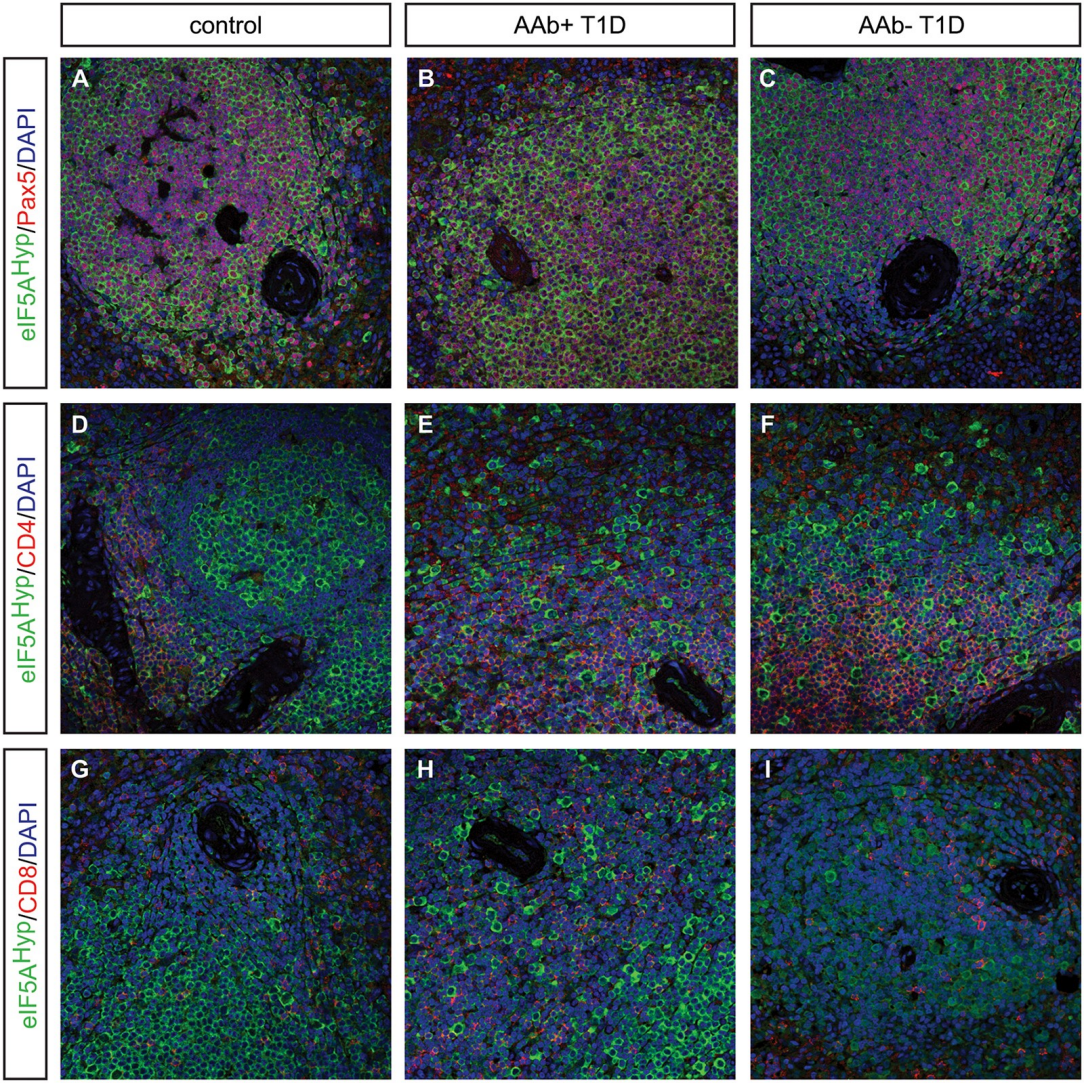
Expression of eIF5A^Hyp^ in spleen tissue from donors with T1D and matched control donors. We examined spleen tissue from persons with autoantibody positive (AAb+) and auto-antibody negative (AAb-) T1D, and corresponding controls matched for age, gender, ethnicity, and BMI. As observed in T2D and matched control spleen tissue, most eIF5A^Hyp^-expressing cells were Pax5+ (A-C); however, some eIF5A^Hyp^+ cells expressed either CD4+ (D-F) or CD8+ (G-I).

Donor pancreas and spleen tissue from individuals with T1D were also acquired from nPOD and evaluated for the expression pattern of eIF5A^Hyp^. This cohort of samples included T1D donors that were autoantibody-positive and autoantibody-negative, with both short and long disease duration; non-diabetic controls were matched for age, gender, ethnicity and BMI (Table 3). Similar to the T2D/control samples, we identified cells co-expressing the hormone PP with high intensity eIF5A^Hyp^ immunostaining (Fig 5A–5F); robust co-expression of eIF5A^Hyp^ with other islet hormones was not observed. Moreover, the eIF5A^Hyp^-expressing cells expressed ChromograninA (Fig 5G–5I), which again confirmed that these cells are neuroendocrine in nature. Evaluation of spleen tissue for all T1D donors and controls revealed an identical pattern of expression to that observed in the T2D donors and controls. Specifically, the majority of eIF5A^Hyp^-expressing cells co-expressed Pax5 (Fig 6; S8–S10 Figs).

## Discussion

Previous data from mouse models identified that pharmacological modulation of the hypusination of eIF5A enhanced beta cell mass and improved glucose tolerance in mouse models of both T1D and T2D [14,16], thereby suggesting an important role for eIF5A^Hyp^ in the setting of diabetes. However, to translate these findings to human, a greater understanding of eIF5A^Hyp^ in the human pancreas and spleen would be required. This study represents the first description of eIF5A^Hyp^ expression in human organs from donors with and without diabetes. Importantly, our results reveal a heretofore unappreciated enrichment of eIF5A^Hyp^ in subsets of endocrine cells in the pancreas and immune cells in the spleen. Interestingly, the presence of eIF5A^Hyp^ co-expressing cells was not obviously enhanced in diseased tissue; however, larger cohorts are required where tissue can be sampled from across whole organs in order to precisely quantitate the presence of these cells and definitively determine correlation with disease.

Further investigation is also required as to the relative abundance of the deoxyhypusine and hypusine forms of eIF5A in both the normal and diseased setting. Currently, published work suggests that the deoxyhypusine form of eIF5A is transient and reversible, and therefore not as abundant due to its rapid modification by the enzyme DOHH (deoxyhypusine hydroxylase) to the hypusinated form of eIF5A during the process of hypusine biosynthesis [25]. Our antibody is specific for the modified forms of eIF5A over the unmodified forms [18], and the form most detectable by our antibody due to its high prevalence is the more stable hypusinated form of eIF5A (eIF5A^Hyp^). Further investigation and tool development will be required to determine and understand the relative abundance of the deoxyhypusine and hypusine forms of eIF5A in both the normal and diseased settings.

Our findings in the pancreas demonstrate that eIF5A^Hyp^ is expressed in both the exocrine and endocrine compartments in mouse and human. Previous reports have also shown expression of eIF5A^Hyp^ in mouse islets [10,11]. However, our immunoblot of sorted mouse islet cells further defined that eIF5A^Hyp^ expression in the islet can be found in both the beta cell and non-beta cell populations. The non-beta cell populations encompass multiple hormone-expressing cell types, and our immunostaining analysis of mouse tissue clarified that the most robust expression of eIF5A^Hyp^ is in the PP cell population. Given the over-representation of PP cells in the uncinate region of the pancreas [26], we analyzed tissue sections that contained the uncinate region and found that eIF5A^Hyp^ is robustly co-expressed in PP cells of human islets. Despite evidence that PP cells have a critical secretory function in the brain-gut axis [27] and may serve as a regulator of intra-islet secretion [28], the role of PP cells in the context of diabetes has received little attention. From a developmental perspective, PP cells are predominantly derived from the ghrelin-expressing cell lineage found in the embryonic pancreas [29]; however, the function of eIF5A^Hyp^ in the PP cell population postnatally or any function for eIF5A^Hyp^ in the development of PP cells has yet to be elucidated. Interestingly, expression analysis of 12-lipoxygenase, a factor known to promote inflammation in the setting of diabetes, is also increased in the PP-expressing cell population in pancreas tissue from human donors (collected through nPOD; [30]). Clearly, a greater understanding is required for the role of PP cells in the pathogenesis of diabetes.

Given that much of the published and ongoing work on hypusine biosynthesis in mice has studied eIF5A^Hyp^ in the context of diabetes, we had hypothesized that eIF5A^Hyp^ expression would be identified predominantly in the insulin-producing beta cell population. Our western blot analysis did reveal eIF5A^Hyp^ expression in human islets. Moreover, we observed eIF5A^Hyp^ expression in a purified population of beta cells (Tomato+) from mouse islets. Interestingly, the quantitative nature of western blots indicates that the expression of eIF5A^Hyp^ must be lower in the purified beta cells compared with non-beta cells given that PP cells comprise only a small portion of the Tomato(-) non-beta cell fraction whereas the Tomato(+) fraction is composed exclusively of beta cells, and we see near equivalent expression of eIF5A^Hyp^ in both sorted populations. This finding is consistent with the immunofluorescence data, wherein we identified robust expression of eIF5A^Hyp^ in PP-expressing cells. The lack of observable co-expression of eIF5A^Hyp^ and hormones in all cell types in the islet was unexpected; however, we must consider the possibility that this may be due to the limitations in detection of low protein expression by immunofluorescence. Therefore, and considering all data together, our observations indicate the presence of eIF5A^Hyp^ in both beta cells and non-beta cells, with particularly high expression in one small non-beta cell populations, the PP cells.

Our previous finding that pharmacological inhibition of eIF5A hypusination (using the drug GC7; N1-Guanyl-1,7-diaminoheptane) in NOD mice improved glucose tolerance and preserved beta cell mass [14]. These improvements were also accompanied by reductions in insulitis, which led us to question whether the improvements in beta cell function were due to a direct effect of DHPS inhibition in beta cells, or an indirect effect related to DHPS inhibition in infiltrating immune cells. Our work and that of others suggest a role for eIF5A^Hyp^ and DHPS in promoting T cell and B cell proliferation [14,31,32], which was the basis for our hypothesis that perhaps eIF5A^Hyp^ is differentially expressed in immune cells in individuals with diabetes compared with controls. However, identical expression patterns were noted in all spleen tissue evaluated. The identical expression patterns of eIF5A^Hyp^ between healthy and disease in both the immune cell populations and islet cell populations could also suggest that it is not the abundance of eIF5A^Hyp^ that is critical for promotion of disease. Rather, the presence of eIF5A^Hyp^ facilitating the translation of different mRNAs in the disease setting compared with the healthy setting could drive pathogenesis. Given our recent findings that deletion of *Dhps* in adult mouse beta cells results in reduced diet-induced beta cell proliferation and subsequent glucose intolerance due to altered translation of cyclinD2 [10], we are now investigating the impact of eIF5A^Hyp^ on mRNA translation in other diabetes-related cell populations.

## Supporting information

S1 Fig Source images for western blots of mouse and human pancreas and islets. (A) Immunoblot images show expression of eIF5A^Hyp^, insulin and total eIF5A in cell lysates from mouse whole pancreas and isolated islets. The top portions of these blots were proved with antibodies not related to this study. (B) Total protein expression as visualized by PonceauS staining. (C) Immunoblot images show expression of eIF5A^Hyp^, insulin and total eIF5A in cell lysate from human exocrine tissue and isolated islets. One blot was probed twice, and this second antibody was not related to this study. (D) Total protein expression as visualized by PonceauS staining. The dotted box shows the lanes where the exocrine and islet samples were run; the other samples are unrelated to this study.
(PDF)

S2 Fig Collection of beta cell and non-beta cell populations by FACS. (A) Islets isolated from multiple RIP-cre;R26RTomato mice were pooled together and processed for fluorescence activated cell sorting (FACS). Islet cells were sorted into two populations: Tomato-positive beta cells (R4), and Tomato-negative non-beta cells (R3, islet cells expressing glucagon, somatostatin, ghrelin, and pancreatic polypeptide).
(PDF)

S3 Fig Source images for western blots of FACS sorted mouse islet cell populations. (A) Immunoblot for expression of Pdx1 and eIF5A^Hyp^ in cell lysates from Tomato-negative non-beta cells and Tomato-positive beta cells. (B) Immunoblot for expression of total eIF5A. (C) Total protein expression as visualized by PonceauS staining.
(PDF)

S4 Fig Evaluation of eIF5AHyp expression following treatment with DHPS inhibitor. HEK293T cells were treated with the DHPS inhibitor GC7 (N1-Guanyl-1,7,diaminoheptane) and analyzed for eIF5AHyp expression by immunofluorescence. (A) Control HEK293T cells uniformly expressed eIF5A^Hyp^. (B,C) Treatment with GC7 resulted in reduced expression of eIF5A^Hyp^. (D) A secondary antibody control was also performed to confirm that the observed signal was not an artifact. Mouse pancreatic islets were also treated with GC7 and analyzed for eIF5A^Hyp^ expression by immunofluorescence. (E) Control mouse islets contained cells with both weak and robust expression of eIF5A^Hyp^. (F, G) Islets treated with GC7 showed a reduction in expression of eIF5A^Hyp^. Images are 20X. Inset images are higher magnification of the areas outlined with white boxes.
(PDF)

S5 Fig eIF5A^Hyp^ expression pattern in the Pax5-expressing cell population in spleen tissue of control and T2D. We evaluated the expression of eIF5A^Hyp^ in Pax5-expressing B cells in the spleens of donors with T2D and controls matched for age, gender and BMI. The fluorescent channels have been separated to better display the expression patterns of the Pax5-expressing B cells (A, B), eIF5A^Hyp^-expressing cells (C, D), and the overlap between the Pax5-expressing and eIF5A^Hyp^-expressing populations (E, F). All images are 20X.
(PDF)

S6 Fig eIF5A^Hyp^ expression pattern in the CD4-expressing cell population in spleen tissue of control and T2D. We evaluated the expression of eIF5A^Hyp^ in CD4-expressing T cells in the spleens of donors with T2D and controls matched for age, gender and BMI. The fluorescent channels have been separated to better display the expression patterns of the CD4-expressing T cells (A, B), eIF5A^Hyp^-expressing cells (C, D), and the minimal overlap between the CD4-expressing and eIF5A^Hyp^-expressing populations (E, F). All images are 20X.
(PDF)

S7 Fig eIF5A^Hyp^ expression pattern in the CD8-expressing T cell population in spleen tissue of control and T2D. We evaluated the expression of eIF5A^Hyp^ in CD8-expressing T cells in the spleens of donors with T2D and controls matched for age, gender and BMI. The fluorescent channels have been separated to better display the expression patterns of the CD8-expressing T cells (A, B), eIF5A^Hyp^-expressing cells (C, D), and the minimal overlap between the CD8-expressing and eIF5A^Hyp^-expressing populations (E, F). All images are 20X.
(PDF)

S8 Fig eIF5A^Hyp^ expression pattern in the Pax5-expressing B cell population in spleen tissue of control and T1D. We evaluated the expression of eIF5A^Hyp^ in Pax5-expressing B cells in the spleens of donors with auto-antibody positive (AAb+) and auto-antibody negative (AAb-) T1D, and corresponding controls matched for age, gender, ethnicity, and BMI. The fluorescent channels have been separated to better display the expression patterns of the Pax5-expressing B cells (A—C), eIF5A^Hyp^-expressing cells (D—F), and the overlap between the Pax5-expressing and eIF5A^Hyp^-expressing populations (G—I). All images are 20X.
(PDF)

S9 Fig eIF5A^Hyp^ expression pattern in the CD4-expressing T cell population in spleen tissue of control and T1D. We evaluated the expression of eIF5A^Hyp^ in CD4-expressing T cells in the spleens of donors with auto-antibody positive (AAb+) and auto-antibody negative (AAb-) T1D, and corresponding controls matched for age, gender, ethnicity, and BMI. The fluorescent channels have been separated to better display the expression patterns of the CD4-expressing T cells (A—C), eIF5A^Hyp^-expressing cells (D—F), and the minimal overlap between the CD4-expressing and eIF5A^Hyp^-expressing populations (G—I). All images are 20X.
(PDF)

S10 Fig eIF5A^Hyp^ expression pattern in the CD8-expressing T cell population in spleen tissue of control and T1D. We evaluated the expression of eIF5A^Hyp^ in CD8-expressing T cells in the spleens of donors with auto-antibody positive (AAb+) and auto-antibody negative (AAb-) T1D, and corresponding controls matched for age, gender, ethnicity, and BMI. The fluorescent channels have been separated to better display the expression patterns of the CD8-expressing T cells (A—C), eIF5A^Hyp^-expressing cells (D—F), and the minimal overlap between the CD8-expressing and eIF5A^Hyp^-expressing populations (G—I). All images are 20X.
(PDF)
