# Supplementary material for: Hypusinated eIF5A is expressed in the pancreas and spleen of individuals with type 1 and type 2 diabetes
Source: PLoS One. 2020 Mar 24;15(3):e0230627. doi: 10.1371/journal.pone.0230627 (PMC7092972; doi:10.1371/journal.pone.0230627)
Supplement: S1 Fig — (A) Immunoblot images show expression of eIF5AHyp, insulin and total eIF5A in cell lysates from mouse whole pancreas and isolated islets. The top portions of these blots were proved with antibodies not related to this study. (B) Total protein expression as visualized by PonceauS staining. (C) Immunoblot images show expression of eIF5AHyp, insulin and total eIF5A in cell lysate from human exocrine tissue and isolated islets. One blot was probed twice, and this second antibody was not related to this study. (D) Total protein expression as visualized by PonceauS staining. The dotted box shows the lanes where the exocrine and islet samples were run; the other samples are unrelated to this study. (PDF) [file pone.0230627.s001.pdf]

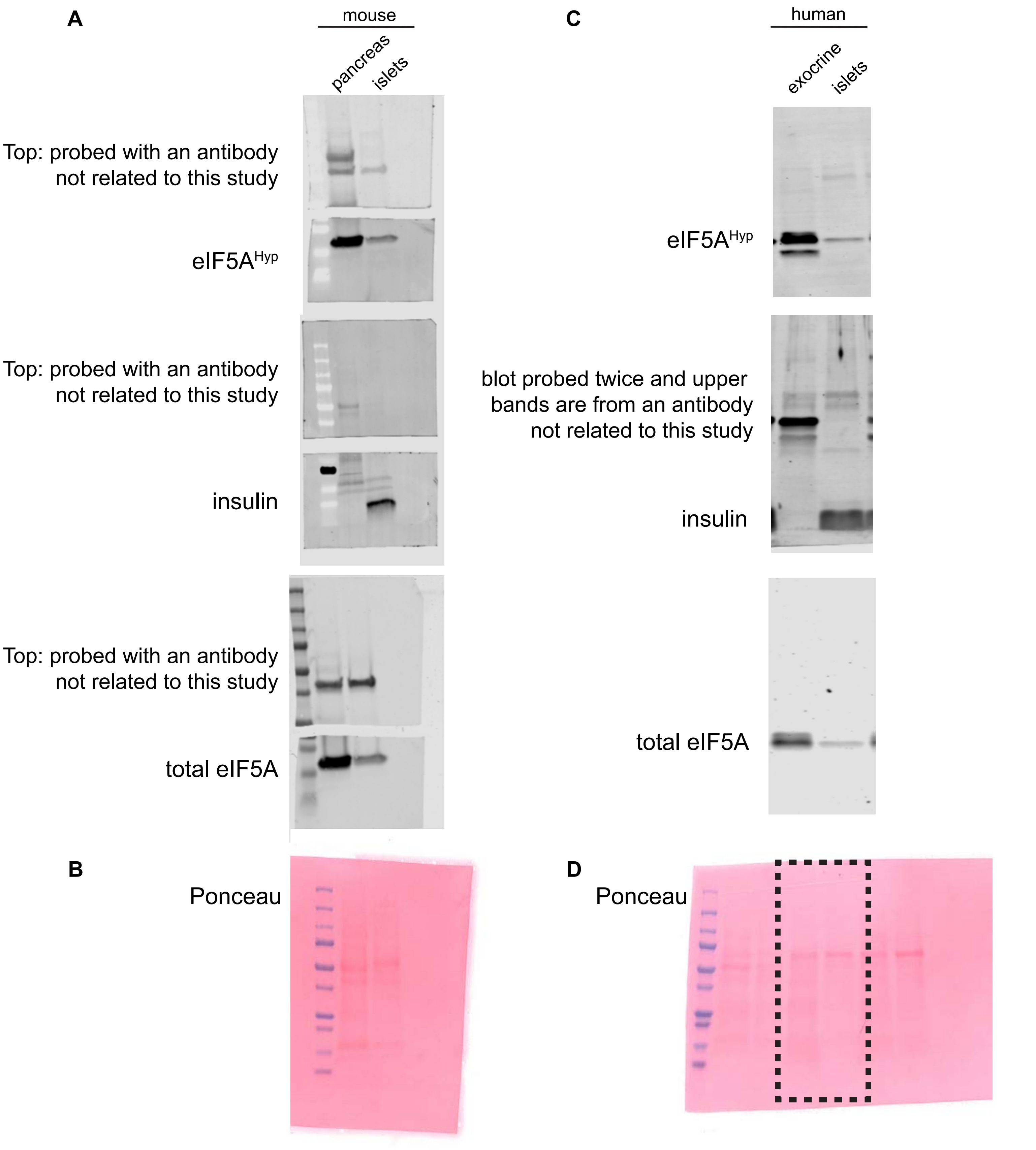

Supplemental Figure 1. Source images for western blots of mouse and human pancreas and islets. (A) Immunoblot images show expression of eIF5A^{Hyp}, insulin, and total eIF5A in cell lysates from mouse whole pancreas and isolated islets. The top portions of these blots were probed with antibodies not related to this study. (B) Total protein expression as visualized by PonceauS staining. (C) Immunoblot images show expression of eIF5A^{Hyp}, insulin, and total eIF5A in cell lysate from human exocrine tissue and isolated islets. One blot was probed twice and this second antibody was not related to this study. (D) Total protein expression as visualized by PonceauS staining. The dotted box shows the lanes where the exocrine and islet samples were run; the other samples are unrelated to this study.
