# Supplementary material for: Hypusinated eIF5A is expressed in the pancreas and spleen of individuals with type 1 and type 2 diabetes
Source: PLoS One. 2020 Mar 24;15(3):e0230627. doi: 10.1371/journal.pone.0230627 (PMC7092972; doi:10.1371/journal.pone.0230627)
Supplement: S2 Fig — (A) Islets isolated from multiple RIP-cre;R26RTomato mice were pooled together and processed for fluorescence activated cell sorting (FACS). Islet cells were sorted into two populations: Tomato-positive beta cells (R4), and Tomato-negative non-beta cells (R3, islet cells expressing glucagon, somatostatin, ghrelin, and pancreatic polypeptide). (PDF) [file pone.0230627.s002.pdf]

A

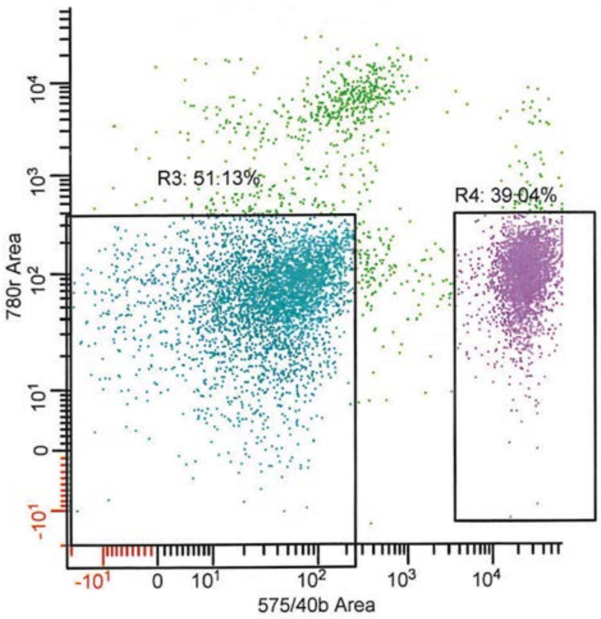

Supplemental Figure 2. Collection of beta cell and non-beta cell populations by FACS.

(A) Islets isolated from multiple *RIP-cre;R26R^{Tomato}* mice were pooled together and processed for fluorescence activated cell sorting (FACS). Islet cells were sorted into two populations: tomato-positive beta cells (R4), and tomato-negative non-beta cells (R3, islet cells expressing glucagon, somatostatin, ghrelin and pancreatic polypeptide).
