# Supplementary material for: Hypusinated eIF5A is expressed in the pancreas and spleen of individuals with type 1 and type 2 diabetes
Source: PLoS One. 2020 Mar 24;15(3):e0230627. doi: 10.1371/journal.pone.0230627 (PMC7092972; doi:10.1371/journal.pone.0230627)
Supplement: S3 Fig — (A) Immunoblot for expression of Pdx1 and eIF5AHyp in cell lysates from Tomato-negative non-beta cells and Tomato-positive beta cells. (B) Immunoblot for expression of total eIF5A. (C) Total protein expression as visualized by PonceauS staining. (PDF) [file pone.0230627.s003.pdf]

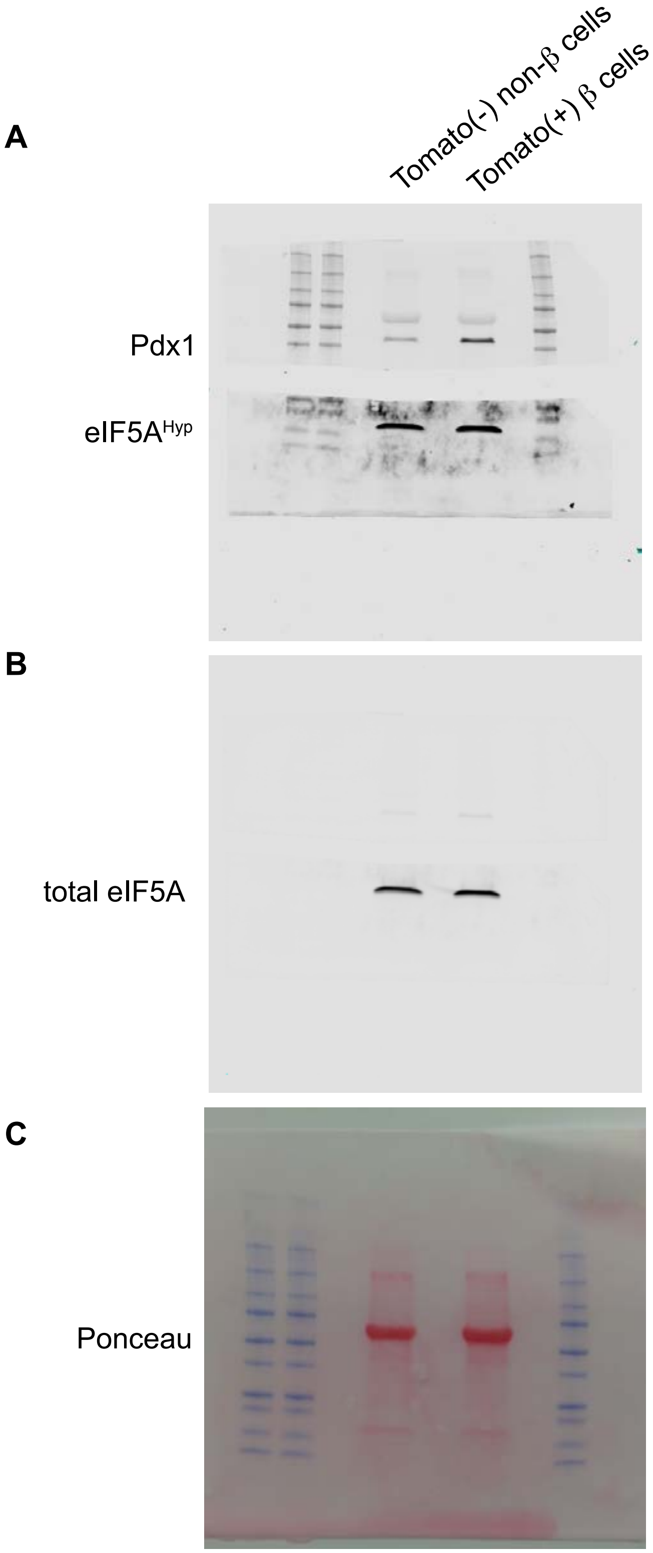

Supplemental Figure 3. Source images for western blots of FACS sorted mouse islet cell populations.

(A) Immunoblot for expression of Pdx1 and eIF5A^{Hyp} in cell lysates from Tomato-negative non-beta cells and Tomato-positive beta cells. (B) Immunoblot for expression of total eIF5A. (C) Total protein expression as visualized using PonceauS staining.
