# Supplementary material for: Hypusinated eIF5A is expressed in the pancreas and spleen of individuals with type 1 and type 2 diabetes
Source: PLoS One. 2020 Mar 24;15(3):e0230627. doi: 10.1371/journal.pone.0230627 (PMC7092972; doi:10.1371/journal.pone.0230627)
Supplement: S4 Fig — HEK293T cells were treated with the DHPS inhibitor GC7 (N1-Guanyl-1,7,diaminoheptane) and analyzed for eIF5AHyp expression by immunofluorescence. (A) Control HEK293T cells uniformly expressed eIF5AHyp. (B,C) Treatment with GC7 resulted in reduced expression of eIF5AHyp. (D) A secondary antibody control was also performed to confirm that the observed signal was not an artifact. Mouse pancreatic islets were also treated with GC7 and analyzed for eIF5AHyp expression by immunofluorescence. (E) Control mouse islets contained cells with both weak and robust expression of eIF5AHyp. (F, G) Islets treated with GC7 showed a reduction in expression of eIF5AHyp. Images are 20X. Inset images are higher magnification of the areas outlined with white boxes. (PDF) [file pone.0230627.s004.pdf]

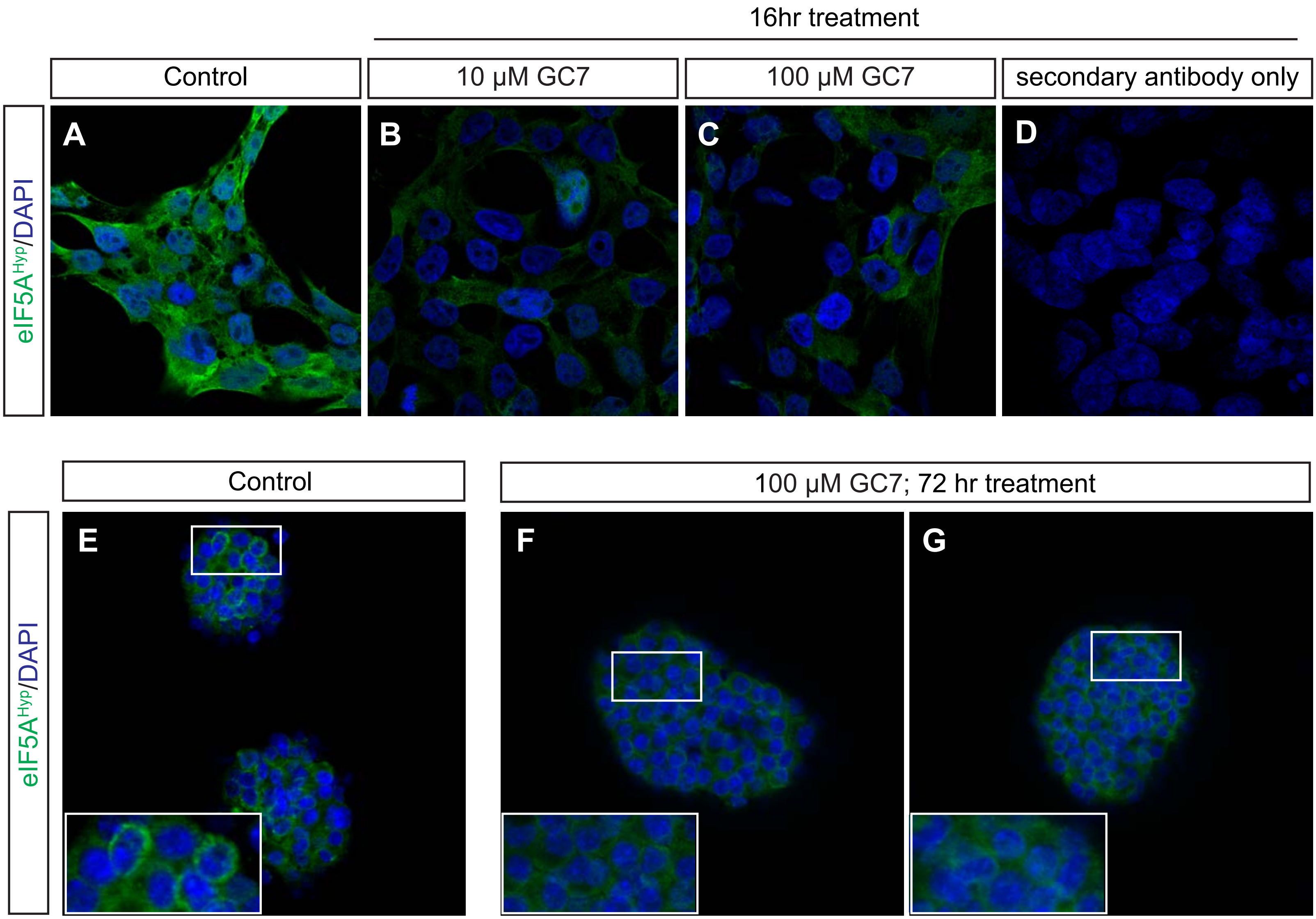

Supplemental Figure 4. Evaluation of eIF5A^{Hyp} expression following treatment with DHPS inhibitor.

HEK293T cells were treated with the DHPS inhibitor GC7 (N1-Guanyl-1,7-diaminoheptane) and analyzed for eIF5A^{Hyp} expression by immunofluorescence. (A) Control HEK293T uniformly expressed eIF5A^{Hyp}. (B, C) Treatment with GC7 resulted in reduced expression of eIF5A^{Hyp}. (D) A secondary antibody control was also performed to confirm that the observed signal was not an artifact. Mouse pancreatic islets were also treated with GC7 and analyzed for eIF5A^{Hyp} expression by immunofluorescence. (E) Control mouse islets contained cells with both weak and robust expression of eIF5A^{Hyp}. (F, G) Islets treated with GC7 showed a reduction in expression of eIF5A^{Hyp}. Images are 20X. Inset images are higher magnification of the areas outlined with white boxes.
