# Supplementary material for: Hypusinated eIF5A is expressed in the pancreas and spleen of individuals with type 1 and type 2 diabetes
Source: PLoS One. 2020 Mar 24;15(3):e0230627. doi: 10.1371/journal.pone.0230627 (PMC7092972; doi:10.1371/journal.pone.0230627)
Supplement: S7 Fig — We evaluated the expression of eIF5AHyp in CD8-expressing T cells in the spleens of donors with T2D and controls matched for age, gender and BMI. The fluorescent channels have been separated to better display the expression patterns of the CD8-expressing T cells (A, B), eIF5AHyp-expressing cells (C, D), and the minimal overlap between the CD8-expressing and eIF5AHyp-expressing populations (E, F). All images are 20X. (PDF) [file pone.0230627.s007.pdf]

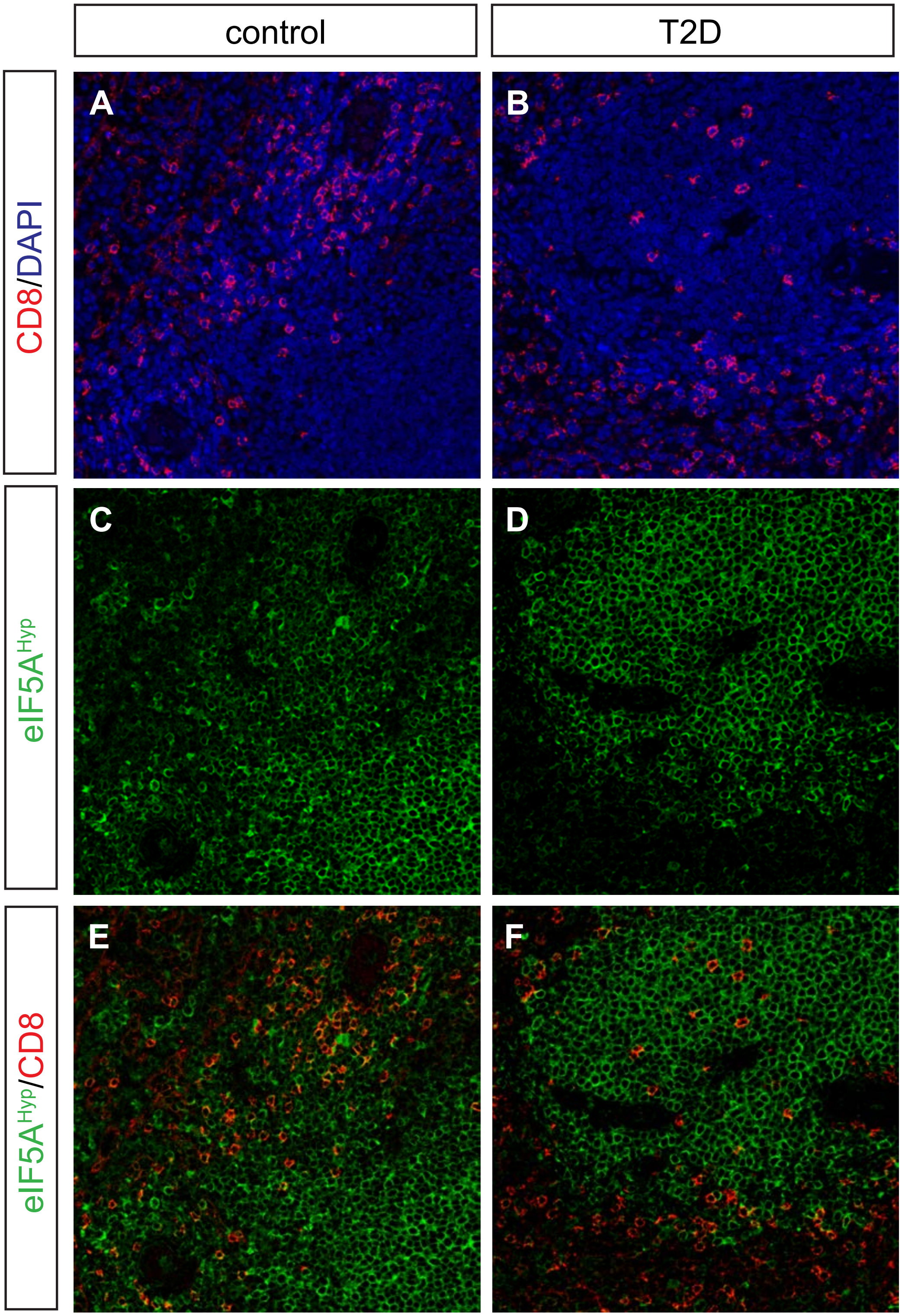

Supplemental Figure 7. eIF5A^{Hyp} expression pattern in the CD8-expressing T cell population in spleen tissue of control and T2D.

We evaluated the expression of eIF5A^{Hyp} in CD8-expressing T cells in the spleens of donors with T2D and controls matched for age, gender and BMI. The fluorescent channels have been separated to better display the expression patterns of the CD8-expressing T cells (A, B), eIF5A^{Hyp}-expressing cells (C, D), and the minimal overlap between the CD8-expressing and eIF5A^{Hyp}-expressing populations (E, F). All images are 20X.
