# Supplementary material for: Hypusinated eIF5A is expressed in the pancreas and spleen of individuals with type 1 and type 2 diabetes
Source: PLoS One. 2020 Mar 24;15(3):e0230627. doi: 10.1371/journal.pone.0230627 (PMC7092972; doi:10.1371/journal.pone.0230627)
Supplement: S8 Fig — We evaluated the expression of eIF5AHyp in Pax5-expressing B cells in the spleens of donors with auto-antibody positive (AAb+) and auto-antibody negative (AAb-) T1D, and corresponding controls matched for age, gender, ethnicity, and BMI. The fluorescent channels have been separated to better display the expression patterns of the Pax5-expressing B cells (A—C), eIF5AHyp-expressing cells (D—F), and the overlap between the Pax5-expressing and eIF5AHyp-expressing populations (G—I). All images are 20X. (PDF) [file pone.0230627.s008.pdf]

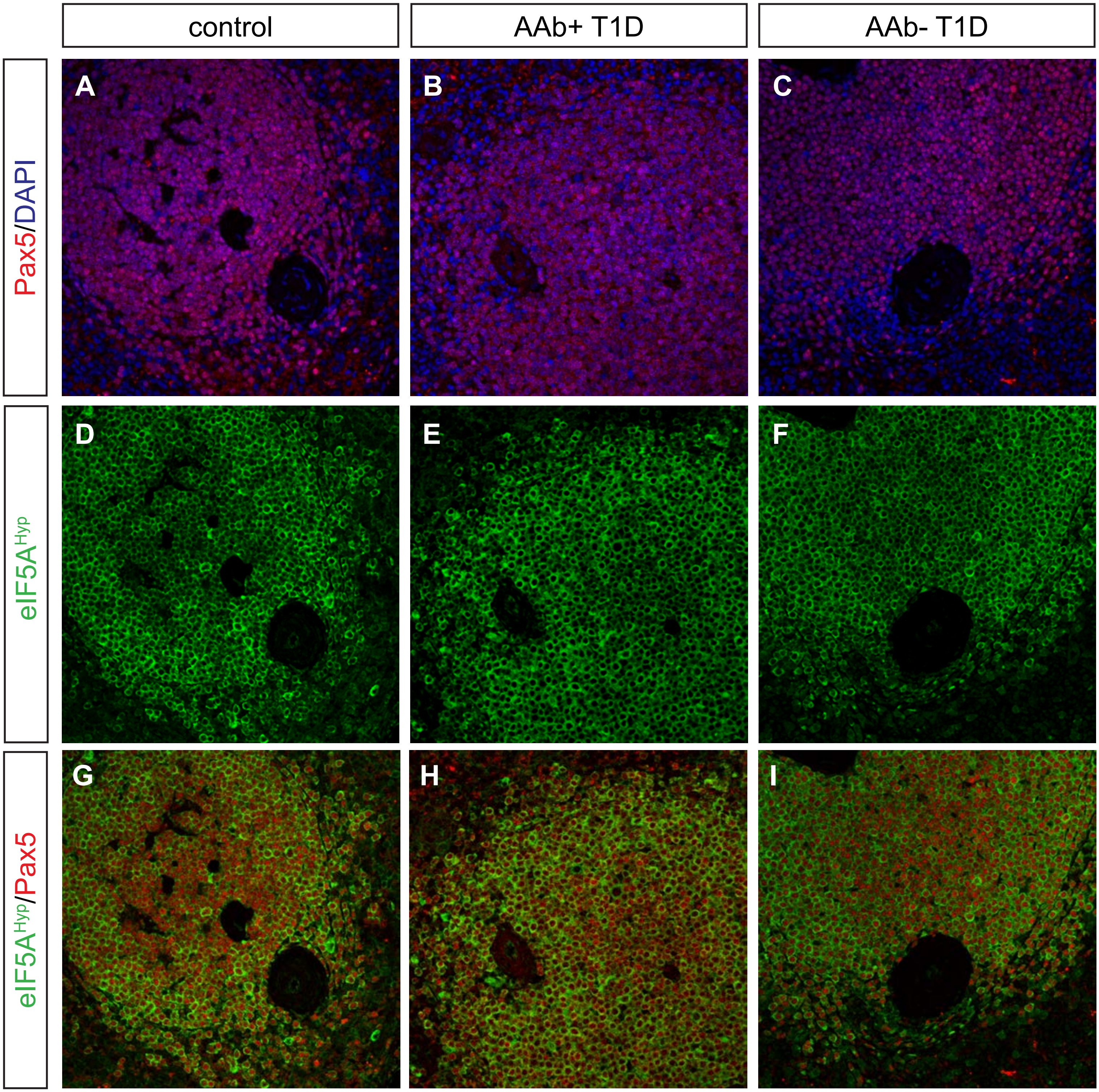

Supplemental Figure 8. eIF5A^{Hyp} expression pattern in the Pax5-expressing B cell population in spleen tissue of control and T1D.

We evaluated the expression of eIF5A^{Hyp} in Pax5-expressing B cells in the spleens of donors with auto -antibody positive (AAb+) and auto-antibody negative (AAb-) T1D, and corresponding controls matched for age, gender, ethnicity, and BMI. The fluorescent channels have been separated to better display the expression patterns of the Pax5-expressing B cells (A - C), eIF5A^{Hyp}-expressing cells (D - F), and the overlap between the Pax5-expressing and eIF5A^{Hyp}-expressing populations (G - I). All images are 20X.
